# Coordination sequences of crystals are of quasi-polynomial type

**DOI:** 10.1107/S2053273320016769

**Published:** 2021-02-18

**Authors:** Yusuke Nakamura, Ryotaro Sakamoto, Takafumi Mase, Junichi Nakagawa

**Affiliations:** a Graduate School of Mathematical Sciences, University of Tokyo, 3-8-1, Komaba, Meguro-ku, Tokyo, 153-8914, Japan; b Department of Mathematics Faculty of Science and Technology, Keio University, 3-14-1 Hiyoshi, Kohoku-ku, Yokohama, 223-8522, Japan

**Keywords:** coordination sequences, graph theory, Hilbert polynomial, monoid theory

## Abstract

The coordination sequence of a graph measures how many vertices the graph has at each distance from a fixed vertex and is a generalization of the coordination number. Here it is proved that the coordination sequence of the graph obtained from a crystal is of quasi-polynomial type, as had been postulated by Grosse-Kunstleve *et al.* [*Acta Cryst.* (1996), A**52**, 879–889].

## Abstract 2

It is proved that the coordination sequence of the graph obtained from a crystal is of quasi-polynomial type, as had been postulated by Grosse-Kunstleve *et al.* [*Acta Cryst.* (1996), A**52**, 879–889] in their study of coordination sequences of zeolites.

## 1. Introduction   

**Figure 1:**
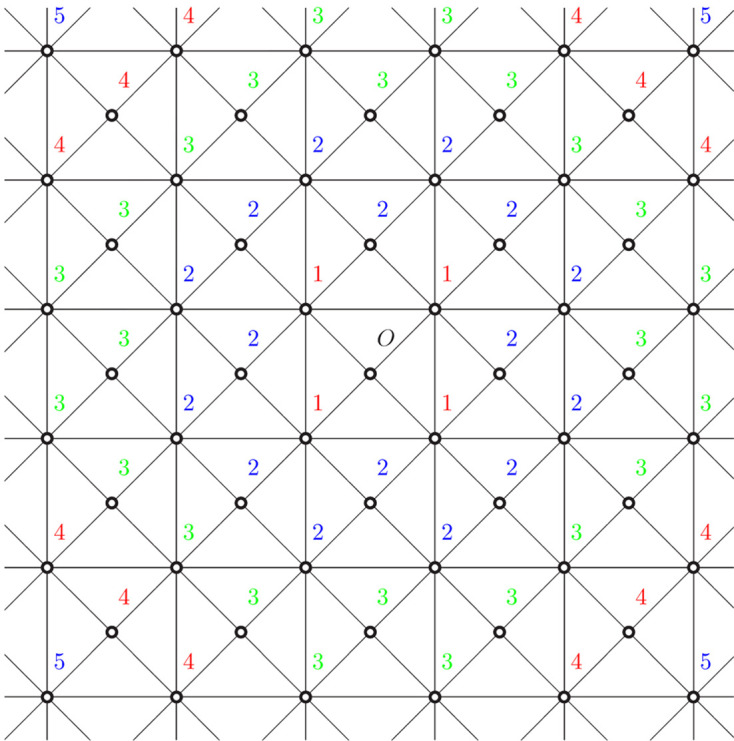
Graph with a 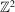 translation symmetry. The number attached to each vertex represents the graph distance from the origin *O*.

For a graph Γ and a fixed vertex 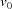 of Γ, and for a non-negative integer *n*, the *coordination sequence* 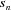 is defined as the number of vertices of Γ at distance *n* from 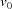. For example, the first few terms of the coordination sequence of the graph in Fig. 1 ▸ are 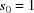, 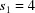, 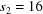, 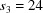. That is, the graph has only one point at distance 0 from the origin (by definition), has four vertices at distance 1, and so on. An easy observation shows that in this case we have 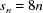 (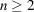).

In this paper, we consider a periodic graph Γ in the following sense:

(A) Γ is a (possibly directed) graph with a free 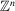 action such that the quotient graph 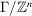 is finite.

This assumption is motivated from the crystallographic viewpoint. Our main example is a graph obtained by a crystal, *i.e.* the vertices of Γ are the set of atoms of the crystal, and to each atomic bond connecting two atoms *u* and *v*, we associate an edge connecting the vertices *u* and *v*. Then, the number 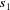 is nothing but the usual coordination number and the coordination sequence 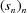 can be thought of as its generalization.

The coordination sequences of periodic graphs are predicted to be of quasi-polynomial type (see Definition 1.2[Statement definition1.2]) by Grosse-Kunstleve *et al.* (1996 ▸). After that, various mathematical methods to calculate coordination sequences have been developed and they are actually calculated in many specific cases as in the work of Conway & Sloane (1997 ▸), Eon (2002 ▸, 2012 ▸), Goodman-Strauss & Sloane (2019 ▸), O’Keeffe (1995 ▸, 1998 ▸), Shutov & Maleev (2018 ▸, 2019 ▸, 2020 ▸).

The purpose of this paper is to give the affirmative answer to the question posed by Grosse-Kunstleve *et al.* (1996 ▸) (see Theorem 2.2[Statement theorem2.2] for the more general statement).

Theorem 1.1 The coordination sequence of a graph satisfying the condition (A) is of quasi-polynomial type. In particular, its generating function is rational. Furthermore, it becomes of polynomial type if the quotient graph 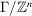 has only one vertex.

Here, we recall the definition of functions of quasi-polynomial type.

Definition 1.2 A function 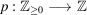 is called a *quasi-polynomial* if there exist an integer 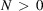 and polynomials 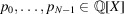 with the following condition:

(i) For any 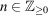, the equality 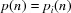 holds if *N* divides 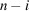.

We say that a function 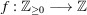 is of *quasi-polynomial type* if there exist an integer 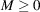 and a quasi-polynomial 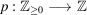 such that 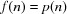 holds for any integer 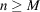. As a special case, we say that *f* is of *polynomial type* when 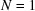.

The paper is organized as follows: in Section 2, we prove Theorem 1.1[Statement theorem1.1] in more general settings (Theorem 2.2[Statement theorem2.2]). The key ingredient of the proof of Theorem 2.2[Statement theorem2.2] is monoid theory in abstract algebra. Readers unfamiliar with monoid theory are advised to read Appendix *A* [App appa] first, where we summarize definitions and propositions in monoid theory with typical examples. In Appendix *A* [App appa], we also prove Theorem A12[Statement theorema12], which is a key to the proof of Theorem 2.2[Statement theorem2.2]. In Section 3, we explain how to apply Theorem 2.2[Statement theorem2.2] to the graph obtained from a crystal, and we give some examples.

## 2. Coordination sequences of graphs with abelian group action   

In this paper, a *graph* 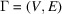 means a directed simple graph, *i.e. V* is the set of vertices and 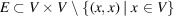 is the set of edges. We say that a graph 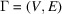 is *finite* when both *V* and *E* are finite sets.

Remark 2.1 A simple undirected graph can be regarded as a graph in the sense above. In fact, since a simple undirected graph 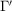 consists of sets 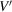 and 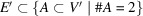, we obtain a graph Γ from 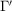 by setting 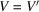 and 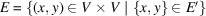. Here the symbol 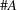 is employed to denote the cardinality of the set *A*.

For vertices 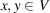, we denote by 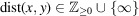 the length of a shortest directed path in *E* from *x* to *y*. If there is no directed path connecting *x* and *y*, then the distance 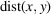 is defined as infinite.

Let *H* be a group. We say that *H acts* on 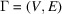 when *H* acts on both *V* and *E* and these actions are compatible, *i.e.* we assume that *H* acts on *V* and the action preserves the adjacency. We then obtain the *quotient graph* 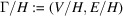. We say that an *H*-action on Γ is *free* when any element of *H*, except for the unit, does not fix a vertex. For more detail, we refer the reader to Eon (2012 ▸).

Theorem 2.2 Let 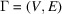 be a graph and let 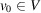 be a vertex. Suppose that an abelian group *H* acts freely on Γ and its quotient graph 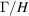 is finite. Then the function 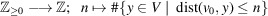 is of quasi-polynomial type. Hence, its difference 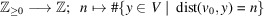
is also of quasi-polynomial type. In particular, their generating functions are rational. Moreover, both the functions are of polynomial type if 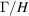 has only one vertex.

Remark 2.3 It is worth emphasizing that we consider not only an undirected graph, but also a directed graph. Hence Theorem 2.2[Statement theorem2.2] can be used for a graph whose edges have a direction. See Example 3.5[Statement example3.5] for a concrete example.

First, we may assume that there exists an abelian group *G* such that *H* is a subgroup of *G* and 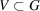 as a set. In fact, since the *H*-action on *V* is free, we have a bijective map 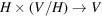 and we can identify 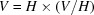 as sets. Let 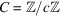 be the cyclic group with order 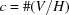. Then, we can identify 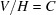 as sets, and hence we may take 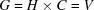.

Remark 2.4 We note that if 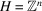 as in Theorem 1.1[Statement theorem1.1], then we may also take 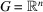. Indeed, for 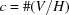, if 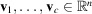 satisfy 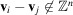 for any 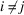, then *V* can be realized as 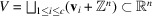. Here we use the symbol 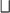 to denote the disjoint union of sets.

We regard the abelian group *G* as an additive group, and denote by 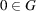 the identity element of *G*. For subsets 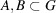 and an element 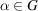, we put 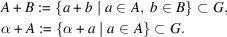
Since 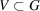, we may assume that 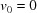 by translation. Since 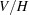 is a finite set, there is a finite subset *F* of *V* such that 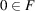 and 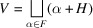
holds.

Definition 2.5 (1) For any two elements 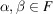, we put 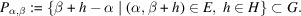
Since the map 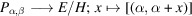 is injective, the finiteness of the set 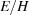 implies that the set 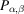 is finite.

(2) For elements 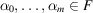 with 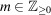, we put 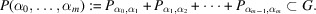
By convention, we define 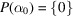 when 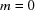. Note that 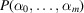 is also a finite set since each 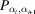 is a finite set.

Remark 2.6 Let 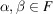. We say that a vertex 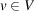 is of α-type if there is an element 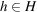 such that 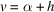. For an α-type vertex 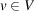 and 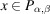, the vertex 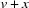 is of β-type and 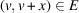. In other words, 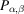 is the set of trans­lations from an α-type vertex *v* to a β-type vertex connected to *v*. Therefore, for an 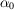-type vertex 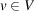 and 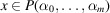, the vertex 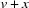 is of 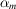-type and there is a path from *v* to 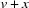 of length *m*. For a concrete example of 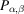 and 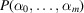, we refer the reader to Example 3.3[Statement example3.3].

Lemma 2.7 For elements 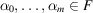 with 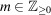, it follows that 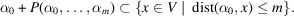

Proof This lemma is obvious by definition (*cf*. Remark 2.6[Statement enun2.6]).

□

For a subset 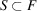, we define a monoid 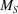 as a submonoid of 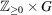. We note that 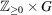 admits a monoid structure by regarding 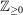 and *G* as monoids by their addition [*cf*. Example A3(3)[Statement examplea3]].

Definition 2.8 Let *S* be a subset of *F*. We define 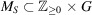 as the submonoid of 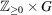 generated by the elements in the set 
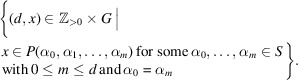
Note that 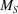 admits a graded monoid structure by the first projection 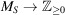, *i.e.* the degree of 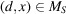 is defined to be *d* [*cf*. Definition A9(1)[Statement definitiona9]].

Remark 2.9 For a generator 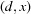 of 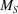, there exists a path 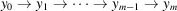
on Γ of length 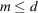 such that 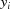 is of 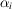-type for some 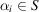, 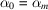 and 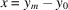. Although the sum of generators is simply defined by the addition of 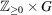, it is not possible in general to interpret the sum as the procedure to connect paths, even if one is allowed to translate each path and to change the order of segments at will. For example, if 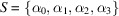, 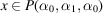 and 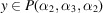, then 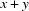 does not always correspond to a path on Γ. See Example 3.3[Statement example3.3] for a concrete example.

Lemma 2.10 For a non-empty subset *S* of *F*, the monoid 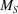 is finitely generated. More precisely, 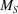 is generated by elements with degree at most 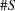.

Proof Take an element 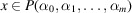 for some 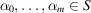 with 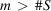 and 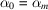. Since 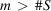, one has 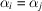 for some 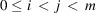, and hence 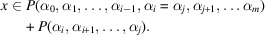
This fact shows that 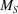 is generated by elements with degree at most 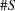. Since there exist only finitely many such elements, 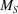 is finitely generated.

□

Next, for a subset 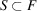 and 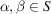, we define 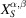 as a subset of 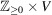, which is proved to be a finitely generated 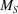-module in Lemma 2.13[Statement lemma2.13].

Definition 2.11 (1) For any two elements 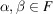, we define the set 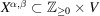 by 
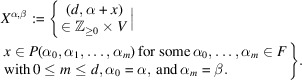

(2) Let *S* be a non-empty subset of *F* and 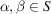. We define the set 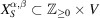 by 
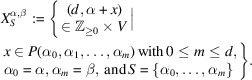

(3) For a subset 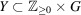 and 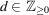, we define 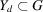 by 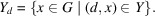

Remark 2.12 (1) By definition, for an element 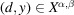, the vertex *y* is of β-type and there is a path from α to *y* of length at most *d*. In other words, the set 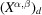 consists of the β-type vertices whose distance from α is less than or equal to *d*.

(2) If a β-type vertex *y* is in 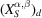, then we have a special path from α to *y*, namely, there exists a path 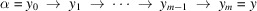
of length 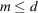 such that 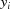 is of 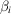-type for some 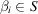 and 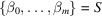. Note that this special path must visit γ-type vertices at least once for each γ in *S*, which plays an essential role in the proof of Lemma 2.13[Statement lemma2.13]. The set 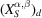 consists of the β-type vertices with such a special path. See Example 3.3[Statement example3.3] for a concrete example.

Lemma 2.13 Let *S* be a non-empty subset of *F* and let 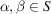. Then, 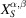 is a finitely generated graded 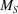-module, where the graded structure of 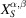 is induced by the first projection 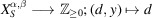.

Proof First, we shall prove that 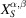 is a graded 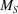-module, *i.e.* 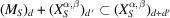 holds for 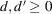 [*cf*. Definition A9(2)[Statement definitiona9]]. Since each element of 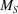 can be written as the sum of generators of the form in Definition 2.8[Statement definition2.8], it is sufficient to show that any generator 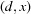 of 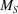 of the form in Definition 2.8[Statement definition2.8] and any element 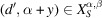 satisfy 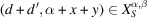. Since 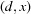 is a generator of the form in Definition 2.8[Statement definition2.8], we have 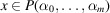 for some 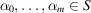 with 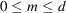 and 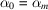. Moreover, by the definition of 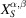, we have 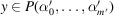 for some 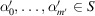 with 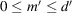 satisfying 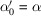, 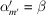 and 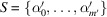.

Since 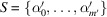, there exists 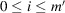 such that 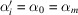. Then, we obtain 
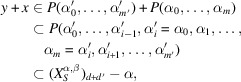
which proves 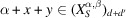. Hence, we have 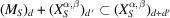 and thus 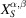 is a graded 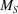-module.

Next we shall see that 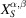 is generated by elements with degree at most 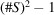. Let 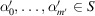 be a sequence with 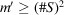, 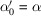, 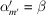 and 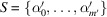. Since 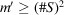, there exist 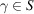 and a subset 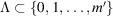 such that 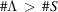 and 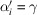 holds for any 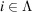.

Here we claim that there exist 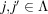 with 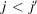 such that 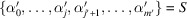.

Let 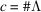 and let 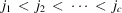 be the elements of Λ. Since 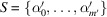, for each 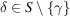, we can take 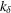 with 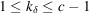 such that δ appears among 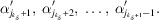
Since 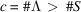, we can take 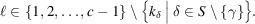
Then 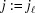 and 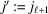 satisfy the claim.

Then the inclusion 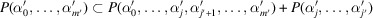
shows that an element of 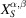 of degree larger than 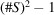 can be written by the sum of an element of 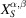 of lower degree and an element of 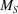. Therefore, 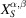 is generated by elements with degree at most 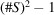. Since there exist only finitely many such elements, 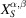 is finitely generated.

□

Lemma 2.14 Let 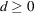 be an integer. Then the following claims are valid.

(1) One has 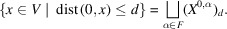

(2) For any element 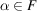, one has 
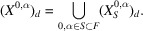
Here *S* runs over the subsets of *F* containing 0 and α.

Proof Claim (2) is obvious by definition. We only give a proof of claim (1). The inclusion 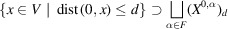
follows from Lemma 2.7[Statement lemma2.7]. Let us show the opposite inclusion. Take an element 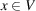 with 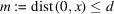. By the definition of the distance, there are edges 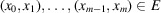 with 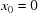 and 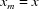. For each *i*, the unique element 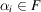 is determined by 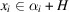. Then 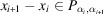, and so one has 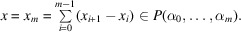
Since 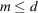, we conclude 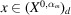.

□

Proof of Theorem 2.2 For an element 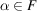, put 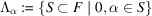 and write 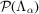 for its power set. Then Lemma 2.14[Statement lemma2.14] and the inclusion–exclusion principle imply 
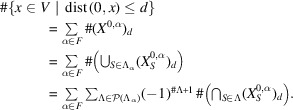
Hence it is enough to show that the function 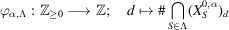
is of quasi-polynomial type.

Lemma 2.10[Statement lemma2.10] and Proposition A6(3)[Statement propositiona6] show that 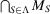 is a finitely generated graded monoid. Furthermore, Lemma 2.13[Statement lemma2.13] and Theorem A12[Statement theorema12] show that 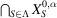 is a finitely generated graded (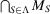)-module. Hence Proposition A11[Statement propositiona11] implies that the function 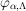 is of quasi-polynomial type, which completes the proof of the first assertion.

By Lemma 2.10[Statement lemma2.10], the monoid 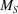 is generated by elements of degree one if 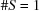. Therefore if 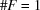, the functions in Theorem 2.2[Statement theorem2.2] turn out to be actually of polynomial type, which completes the proof of the second assertion.

□

Remark 2.15 It is natural to ask how to calculate the quasi-polynomials in Theorem 2.2[Statement theorem2.2] from the graph Γ in some concrete situations.

By the argument in this section, in order to determine the quasi-polynomials, it is sufficient to determine the Hilbert polynomial of 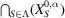 for each Λ. If we know generators of the monoid 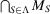 and the module 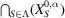, then the Hilbert polynomial of 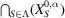 can be calculated in principle via a free resolution (*cf*. Bruns & Herzog, 1993 ▸, Lemma 4.1.13), and can be computed by the standard computational commutative algebra packages *Singular* and *Macaulay2*. Actually in Example 3.1[Statement example3.1], Example 3.2[Statement example3.2] and the face-centered cubic system in Example 3.4[Statement example3.4], generators of 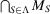 and 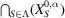 are easily computed. Therefore, in these cases, it is not hard to calculate their Hilbert polynomials from the material in this section. It should be noted, however, that in many cases, even when generators are known, it is easier to calculate their coordination sequences by predicting the region that consists of the vertices with distance at most *n* and proving it by induction.

Computing generators of 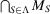 and 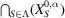 is a more difficult problem in general situations. Indeed, Theorem A12[Statement theorema12] or even its proof does not give a procedure to compute their generators. For example, as we will see later, in the case of Example 3.3[Statement example3.3], computing generators of 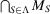 and 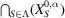 by hand is not easy at all, whereas the graph itself looks simple.

## 3. Examples   

In this section, we explain using some examples how to apply Theorem 2.2[Statement theorem2.2] to the graph obtained from crystals. In Example 3.3[Statement example3.3], we see the complicated notations defined in the previous section. It should be noted in advance, however, that although Theorem 2.2[Statement theorem2.2] guarantees that the coordination sequence of a crystal is of quasi-polynomial type, it is not practical in general to concretely calculate the whole sequence through the theorem or its proof (see Remark 2.15[Statement enun2.15]). Throughout this section, we take *G* as a Euclidean space so that the reader can easily visualize examples.

Example 3.1 One of the simplest examples of crystal structure is the square tiling. Let 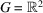, 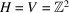 and let 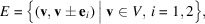
where 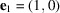 and 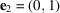. It is obvious that this graph satisfies all the assumptions of Theorem 2.2[Statement theorem2.2]. An easy observation shows that the coordination sequence of this graph from the origin is 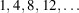, the general term of which can be written as 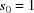, 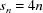 (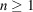).

Let us briefly look at the notations in the previous section. In this case, the finite set *F* used in Section 2 consists of the origin *O*. To be precise, since 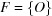, we have 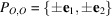. Hence, the 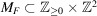 is the graded monoid generated by 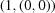, 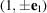 and 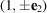, which coincides with the 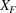 since 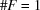.

In contrast, in the case where *F* consists of two or more points, calculating the coordination sequence by this procedure is much more complicated as seen in Remark 2.15[Statement enun2.15] and Example 3.3[Statement example3.3].

**Figure 2:**
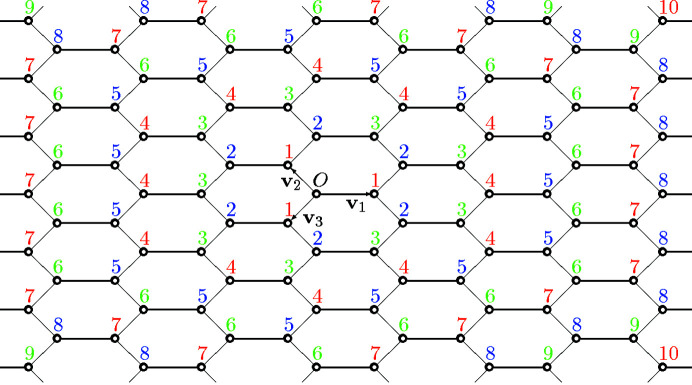
Hexagonal tiling. The number attached to each vertex represents the graph distance from the origin *O*.

Example 3.2 Let 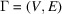 be the graph corresponding to the hexagonal tiling as in Fig. 2 ▸. Let 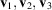 be the vectors corresponding to the edges from *O*, respectively. Note that these vectors satisfy 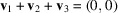. Let 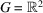 and 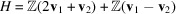. Then, *V* and *E* are *H*-invariant and the quotient graph 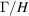 is finite. Since 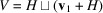, we have 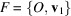. An easy observation shows that the coordination sequence of this graph is 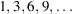, the general term of which can be written as 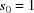, 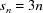 (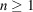).

**Figure 3:**
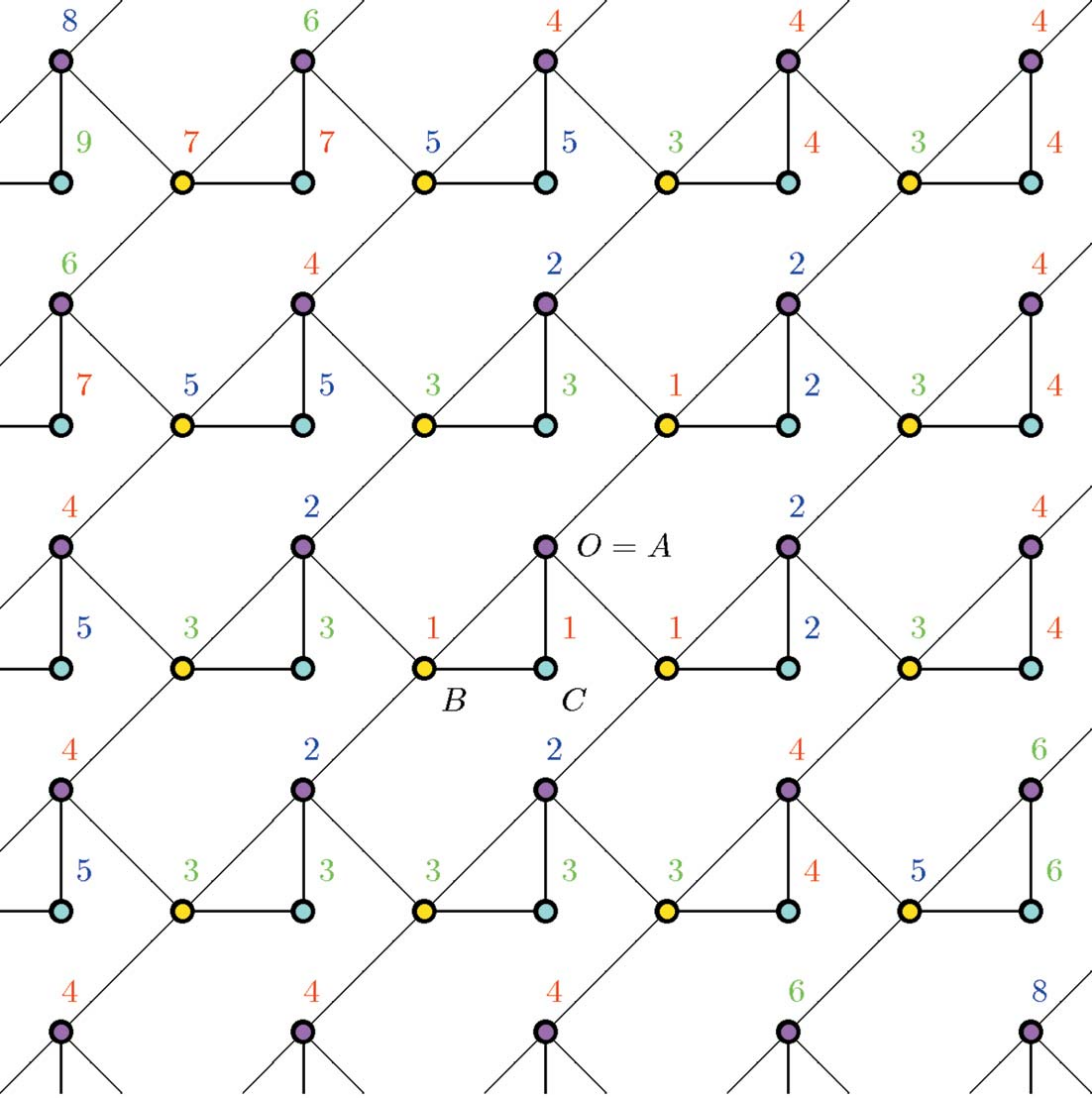
Graph with a 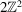-translation symmetry. The number attached to each vertex represents the graph distance from the origin *O*. The color of each vertex represents the equivalence class in 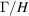.

Example 3.3 Let us see the notations used in Section 2 on an example. Let 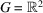 and 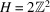. Let 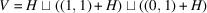
and let 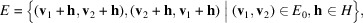
where 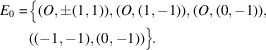
Then, the graph 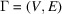 is as in Fig. 3 ▸.

The group *H* acts freely on Γ as translations and the quotient graph 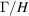 has three vertices. The *F* in Section 2 can be taken as 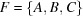, where 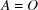, 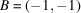, 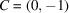. Then, the 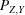 for each 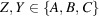 in Definition 2.5[Statement definition2.5] is 
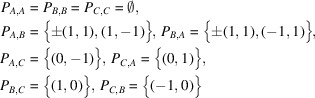
and, for example, one can compute 
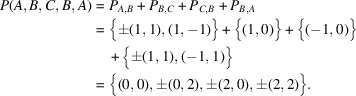
Next, let us see generators of the monoids and the modules in Definition 2.8[Statement definition2.8] and Definition 2.11[Statement definition2.11], respectively. For each subset 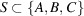 satisfying 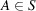, a generator of the monoid 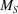 can be computed as 
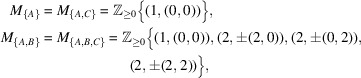

*i.e.* for example, one can take 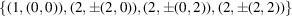
as a generator of the monoid 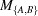. For each subset 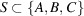 satisfying 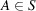 and for each 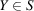, a generator of the 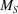-module 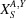 can be taken as 
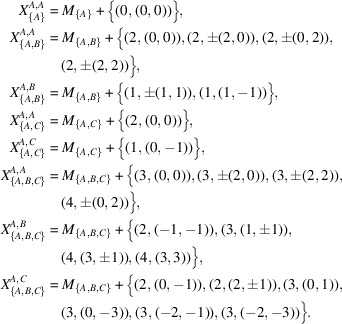
For example, 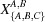 contains 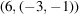, which corresponds to the path 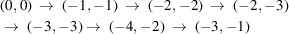
of length 6. Indeed, 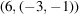 decomposes as 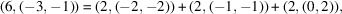
where 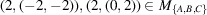 and 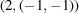 is contained in the generator described above.

Using these data, it is possible in principle to calculate the coordination sequence of the graph. The actual calculation is, however, extremely laborious and shall be omitted in this paper. The coordination sequence of this graph is obtained by Wakatsuki (2018 ▸) as 
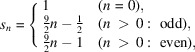
which is not of polynomial type but of quasi-polynomial type.

**Figure 4:**
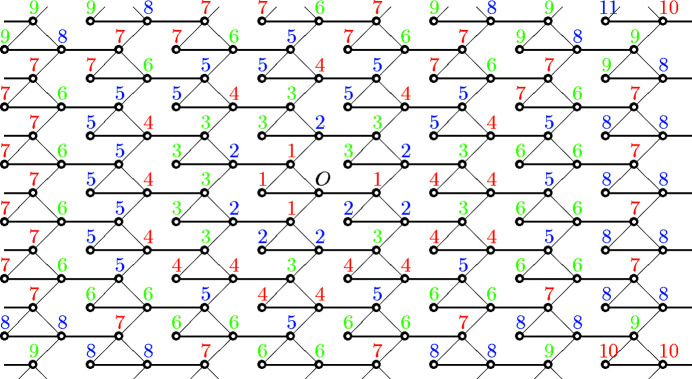
Another realization of the graph in Fig. 3 ▸. This graph can be obtained by adding vertices and edges to the graph in Fig. 2 ▸. If 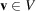 is a vertex of a hexagon, then the graph distance between 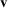 and *O* is exactly the same as that in Example 3.2[Statement example3.2].

Note that Γ has another simple realization on 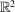 as in Fig. 4 ▸. Since the coordination sequence depends only on its abstract graph structure, these two graphs have the same coordination sequence. As seen in this example, the choice of a realization of a graph is not relevant to Theorem 1.1[Statement theorem1.1].

Example 3.4 Let us consider the graphs corresponding to crystal structures of dimension 3. These graphs have such a translation symmetry that the corresponding quotient graph is finite. Therefore, it follows from Theorem 1.1[Statement theorem1.1] that the coordination sequences of these graphs are of quasi-polynomial type. It should be stressed that, by definition, any crystal structure of any dimension falls into this category.

Let us consider as an example the graph corresponding to the face-centered cubic system. Let 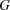 = 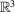, 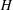 = 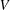 = 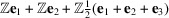 and 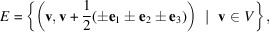
where 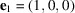, 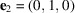, 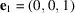 and the signs in the definition of *E* are arbitrary. Then, the graph 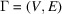 corresponds to the face-centered cubic system. It is clear by definition that *H* acts on Γ and the quotient is finite. Note that while the *H* defined above is the largest translation symmetry of this system, it might be more natural to take 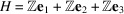 when one considers crystal structure. Even in that case, the graph Γ has an *H*-action and the quotient is finite. As seen in this example, the choice of a unit cell is not relevant to Theorem 1.1[Statement theorem1.1].

Example 3.5 We give one of the simplest examples of a periodic directed graph (one should compare this with Example 3.1[Statement example3.1]). Let 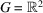, 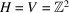 and let 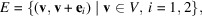
where 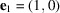 and 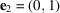. An easy observation shows that the coordination sequence of this graph from the origin is 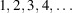, the general term of which can be written as 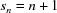 (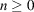).

In this case, the finite set *F* used in Section 2 consists of the origin *O*. To be precise, since 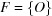, we have 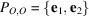. Hence, the 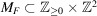 is the graded monoid generated by 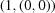, 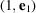 and 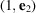, which coincides with the 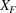 since 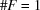.

Example 3.6 We give one of the simplest examples of a periodic graph with *H* having a torsion. Let 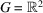 and 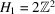. Let 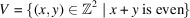
and let 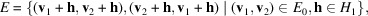
where 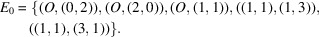
Then, it is obvious that 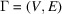 and 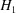 satisfy all the assumptions of Theorem 2.2[Statement theorem2.2]. In this case, we have 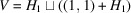 and 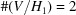.

The graph Γ also admits a 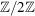-action as follows. Let 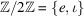, where *e* is the identity element of 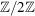. We define a 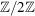-action on *V* by the map 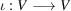 satisfying 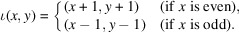
Then, it is easy to see that the set *E* of edges is stable under the 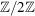-action, and that ι commutes with the 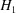-action. Therefore the product 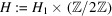 acts on Γ. Then, 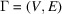 and *H* also satisfy all the assumptions of Theorem 2.2[Statement theorem2.2], and we have 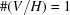 in this case. Therefore, according to Theorem 2.2[Statement theorem2.2], the coordination sequence of this graph should not only be of quasi-polynomial type, but also of polynomial type.

Actually, an easy observation shows that the coordination sequence of this graph from the origin is 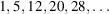, the general term of which can be written as 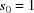, 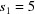, 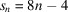 (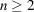).

## 4. Conclusion   

In this paper, we proved that if a graph Γ has a free 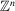-action such that the quotient 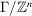 is finite, then the coordination sequence of Γ must be of quasi-polynomial type (Theorem 1.1[Statement theorem1.1] and Theorem 2.2[Statement theorem2.2]). As we mentioned in Example 3.4[Statement example3.4], Theorem 2.2[Statement theorem2.2] can be applied to all crystals, which by definition have such a translation symmetry. It should be noted, however, that except for some simple cases, Theorem 2.2[Statement theorem2.2] or even its proof does not give a specific procedure to concretely calculate coordination sequences (Remark 2.15[Statement enun2.15]). Establishing a systematic method to calculate coordination sequences from an algebraic perspective is left for a future work. The first step would be to determine the period *N* and the number *M* in Definition 1.2[Statement definition1.2]. Once that is done, we can determine the quasi-polynomial by just calculating the first 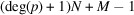 terms.

## Appendix A On finitely generated monoids

In this appendix, we first recall some basic definitions and results on finitely generated monoids [for more detail we refer the reader to Bruns & Gubeladze (2009 ▸)]. Then, we will prove Theorem A12[Statement theorema12], which plays an essential role in the proof of Theorem 2.2[Statement theorem2.2].

### A1. Monoids and modules

In this paper, all the monoids considered are commutative, that is, a *monoid* is a commutative semi-group with the unit element 0, and the operation is written additively.

Definition A1 (1) A *monoid* is a set *M* with a binary operation  with the following three conditions: (i)  holds for . (ii)  holds for any . (iii) There exists an element  such that  holds for any . (2) A monoid *M* is called *integral* when it has the cancelation property, *i.e.* when  implies  for all . (3) A *submonoid* of a monoid *M* is a subset  such that *N* contains the unit of *M* and is closed under the additive operation of *M*. (4) A map  between two monoids is called a *homomorphism* when  and  hold for any . (5) Let *F* be a subset of a monoid *M*. We say that *F generates M* if  holds, *i.e.* any element *m* can be written by

for some ,  and . A monoid *M* is said to be *finitely generated* when *M* is generated by a finite subset of *M*. (6) For an integral monoid *M*, we define the *group of differences*  by  with the following relation ∼: (i)  if and only if . Note that  becomes an abelian group by the addition . Furthermore, we may consider *M* as a submonoid of  by the injective homomorphism .

Remark A2 The notions in (3), (4) and (5) correspond to a subgroup, a group homomorphism and a finitely generated group in group theory, respectively. For a monoid *M* and a field *k*, we can associate the monoid ring  (see Bruns & Gubeladze, 2009 ▸, p. 51 for detail). Then the finite generation of *M* is equivalent to the finite generation of  as a *k*-algebra (*cf*. Bruns & Gubeladze, 2009 ▸, Proposition 2.7).

Example A3 (1) If each element of a monoid *M* has an inverse, then *M* is an abelian group. In particular, any abelian group is an integral monoid. All the monoids appearing in Section 2 are submonoids of abelian groups. Hence they are always integral. (2) The set  of non-negative integers is a typical example of a monoid that is not a group. (3) For monoids *M* and *N*, their Cartesian product  admits a monoid structure by  for . (4) Using the notion of polyhedral convex geometry, we can obtain various non-trivial examples of monoids. A *cone C* in  is the intersection of finitely many linear closed halfspaces (*cf*. Bruns & Gubeladze, 2009 ▸, Definition 1.14). A linear closed halfspace here means a closed halfspace which is defined by a linear function on , *i.e.* it is a set of the form  where . Then  is a submonoid of . We say that a cone *C* is *rational* if *C* is the intersection of finitely many linear closed rational halfspaces, *i.e.* linear closed halfspaces defined by linear functions  with rational coefficients . In this case,  is known to be a finitely generated monoid [see Proposition A8(1)[Statement propositiona8] below]. For example, if

 is finitely generated, but  is not.  is actually generated by ,  and . Furthermore, we have  =  = .

Next we introduce the notation of *M*-modules. It corresponds to the notation of *R*-modules for a commutative ring *R* in ring theory.

Definition A4 Let *M* be a monoid. (1) An *M-module* is a set *X* equipped with an operation , which is written as +, satisfying the following conditions for all  and : (i) , (ii) . (2) Let *F* be a subset of an *M*-module *X*. The module *X* is said to be *generated* by *F* if  holds, where we set

The module *X* is said to be *finitely generated* if some finite subset  generates *X*.

Example A5 (1) In Section 2, we mainly consider the following situation. *M* is a submonoid of an abelian group *G*, and *X* is a subset of *G*. Then *X* is an *M*-module (by the addition of *G*) if and only if  holds. (2) Let *M* be a submonoid of a monoid *N*. Then *N* and *N*-modules can be thought of as *M*-modules. Suppose that *N* is finitely generated as an *M*-module and *X* is a finitely generated *N*-module. Then *X* is finitely generated as an *M*-module. We prove this claim below. Since *N* is finitely generated as an *M*-module, there exists a finite subset  such that . Furthermore, since *X* is finitely generated as an *N*-module, there exists a finite set  such that . Then we have

Since  is a finite set, *X* is finitely generated as an *M*-module. (3) A *polyhedron P* in  is the (possibly unbounded) intersection of finitely many affine closed halfspaces (*cf*. Bruns & Gubeladze, 2009 ▸, Definition 1.1). An affine closed halfspace here means a closed halfspace which is defined by an affine function on , *i.e.* it is a set of the form

for some . Let  be a polyhedron, where  is an affine halfspace. Then the *recession cone*  of *P* is defined by

where  denotes the linear closed halfspace parallel to . Then we have  since  holds for  and  for each *i*. Since , we have

Therefore,  is a -module. We say that a polyhedron  is *rational* if each halfspace  is defined by an affine function  of rational coefficients . In this case,  is known to be a finitely generated -module [see Proposition A8(2)[Statement propositiona8] below]. For example, if

then we have

Therefore we have

It is easy to see that  is generated by (1,3), (2,2) and (3,1) as a -module.

We list some properties on finite generation.

Proposition A6 (1) (Bruns & Gubeladze, 2009 ▸, Proposition 2.8) Let *M* be a finitely generated monoid and let *X* be a finitely generated *M*-module. Then any *M*-submodule of *X* is finitely generated as an *M*-module. (2) (*cf*. Ogus, 2018 ▸, ch. I, Theorem 2.1.17.6) Let  be a homomorphism between integral monoids and let  be a submonoid. If *M* and  are finitely generated, then so is . (3) (*cf*. Bruns & Gubeladze, 2009 ▸, Corollary 2.11) Let *N* be an integral monoid and let  and  be submonoids of *N*. If  and  are finitely generated, then  is also a finitely generated monoid.

Remark A7 Via the correspondence between a monoid *M* and a monoid ring  (*cf*. Bruns & Gubeladze, 2009 ▸, Proposition 2.7), (1) is a corollary of the Hilbert basis theorem in ring theory, which states that a finitely generated *k*-algebra is Noetherian (*cf*. Eisenbud, 1995 ▸, ch. I, Section 1.4). In Ogus (2018 ▸, ch. I, Theorem 2.1.17.6), (2) is proved in a more general setting: (4) Let  and  be monoid homomorphisms. If the monoids *M* and *P* are finitely generated and *N* is integral, then their fiber product  is a finitely generated monoid. We note that the fiber product is defined as

and it is easy to see that  is a submonoid of . (2) can be seen as a special case of (4) by setting *g* as the inclusion map . We note that in Bruns & Gubeladze (2009 ▸, Corollary 2.11), (3) is proved only when . In general cases, the assertion follows from (2), applying it to the inclusion map  and . We also note that in Bruns & Gubeladze (2009 ▸), a monoid *M* is called *affine* when *M* is finitely generated and is isomorphic to a submonoid of .

If a cone and a polyhedron are rational [see Example A3(4)[Statement examplea3] and Example A5(3)[Statement examplea5]], then their restrictions to  give a finitely generated monoid *M* and a finitely generated *M*-module. In (3), we denote by  the cone which consists of the elements of the form  with ,  and .

Proposition A8 (1) (Bruns & Gubeladze, 2009 ▸, Lemma 2.9) For a rational cone , the monoid  is finitely generated. (2) (Bruns & Gubeladze, 2009 ▸, Theorem 2.12) For a rational polyhedron , the set  is a finitely generated ()-module. (3) (Bruns & Gubeladze, 2009 ▸, Corollary 2.10) If *M* is a finitely generated submonoid of , then the monoid  is finitely generated as an *M*-module. (4) [Bruns & Gubeladze, 2009 ▸, Proof of Corollary 2.11(a)] If *M* and *N* are submonoids of , then  =  holds.

In the rest of this section, we summarize the fact on the Hilbert function of graded monoids.

Definition A9 (1) A (positively) *graded monoid* is a monoid *M* equipped with a monoid homomorphism . We say that  is *of degree i* if . Furthermore, we write . (2) Let *M* be a graded monoid. A *graded *M*-module* is an *M*-module *X* equipped with a map  with the condition  for all  and , where we set . This condition is equivalent to saying that  holds for any  and .

Definition A10 Let *M* be a graded monoid and *X* a graded *M*-module with  for any . The function

is called the *Hilbert function* associated with *X*. Its generating function

is called the *Hilbert series* of *X*. The Hilbert function and the Hilbert series depend on the grading of *M* and *X*.

Proposition A11 (Bruns & Gubeladze, 2009 ▸, Theorems 6.38 and 6.39; Bruns & Herzog, 1993 ▸, Theorem 4.1.3, Proposition 4.4.1, Theorem 4.4.3) Let *M* be a graded monoid which is generated by finitely many elements of positive degree. Let *X* be a finitely generated graded *M*-module. Then the Hilbert series  is a rational function, and the Hilbert function  is of quasi-polynomial type. Moreover, if *M* is generated by elements of degree one, then  is of polynomial type.

We note that the assumption  in Definition A10[Statement definitiona10] is satisfied under the assumption of Proposition A11[Statement propositiona11].

### A2. Finite generation of modules

The following theorem is the main theorem in this appendix.

Theorem A12 Let  and  be finitely generated sub­monoids of an integral monoid *M* and let  be a finitely generated -submodule for . Then the -module  is finitely generated.

Proof Since  is a finitely generated -module, there is a finite subset  of  such that , and so one has

Hence by replacing  with , we may assume that  is the form of  for each . Next, replacing *M* with , we may assume that *M* is an abelian group. Furthermore, replacing *M* with the monoid generated by  and the elements of , we may assume that *M* is finitely generated as a monoid. Let  be a generator of *M*. Then, one can take a surjective homomorphism:

Then, the monoid  is finitely generated by Proposition A6(2)[Statement propositiona6]. Take an element  satisfying . Since , we have

Hence Proposition A6(1)[Statement propositiona6] shows that the -module  is finitely generated, and hence its translation  is also a finitely generated -module. Moreover, we have

since φ is surjective. Thus, in order to prove that  is finitely generated as a -module, it is sufficient to show that  is finitely generated as a -module. Since we have

the proof is completed if we show the theorem in the case of . The set  is a rational polyhedron satisfying . Proposition A8(2)[Statement propositiona8] implies that the set

is a finitely generated -module. Since  and  are finitely generated monoids, so is the monoid  by Proposition A6(3)[Statement propositiona6]. Furthermore,

holds by Proposition A8(4)[Statement propositiona8], and it is a finitely generated -module by Proposition A8(3)[Statement propositiona8]. Hence *Y* is a finitely generated -module [*cf*. Example A5(2)[Statement examplea5]]. Since  is an -submodule, it is a finitely generated -module by Proposition A6(1)[Statement propositiona6], which completes the proof. □
